# A Concept Analysis of the Social Responsibility of Nursing Organizations Based on Walker and Avant’s Method

**DOI:** 10.3390/nursrep13040123

**Published:** 2023-10-17

**Authors:** Jong Gun Kim

**Affiliations:** Department of Nursing Science, Hoseo University, Asan 31499, Republic of Korea; jaykim134@hoseo.edu

**Keywords:** social responsibility, nursing organization, concept analysis

## Abstract

Social responsibility has been a core value of the nursing profession, particularly in the area of health disparity. Nevertheless, it is unclear what is meant by social responsibility. This study examined ways to define the concept of the social responsibility of nursing organizations to understand the meaning of social responsibility in the nursing profession. Methods: The concept analysis process reported by Walker and Avant was used to clarify the meaning of social responsibility in nursing organizations. Results: Defining the attributes of the social responsibility of nursing organizations included accessing, educating, and practicing as approaches for strategizing the social and structural change in inequity, caring for oppressed groups suffering socially from those with privilege and power, and taking action for health policy changes in social and political unequal contexts. The antecedents of social responsibility in nursing organizations included recognizing personal characteristics, the perspective of vulnerable populations, and the social and environmental status quo, as well as educating public services on the ethical and moral reasoning of social issues. The consequences of the social responsibility of nursing organizations were achieving social justice as equal access to basic human health needed at a societal level, equal access to effective nursing practices, and the development of health promotion policies for world health administrative practices in nursing. Conclusion: This study provides guidance to direct future studies by identifying conceptual attributes in the context of the social responsibility of nursing organizations.

## 1. Necessity of Research

The concept of social responsibility in nursing organizations, such as nursing schools, hospitals, and nursing institutions, is in their core values, curricular design, admission standards, clinical practice, and service learning opportunities [1,2,3,4,5]. Not just in the field of nursing, the International Organization for Standardization (ISO), the world’s largest organization that develops and unifies international standards, has published international standards for “SR: Social Responsibility ISO 26000” covering the social responsibilities of organizations such as businesses, universities, government agencies, and private organizations [6]. Hence, organizations should assume economic and legal responsibilities to stakeholders and actively carry out a wide range of social responsibilities. The concept of corporate social responsibility was first introduced in the 1950s, but it is only recently that its importance has been highlighted in corporate macro-management strategies [7]. The interest in corporate social responsibility began when it was accepted as a core task of corporate management that companies must perform, and this has become a critical factor in enhancing the corporate image [7]. In addition, the demands of the members of an organization due to social diversification are becoming more diverse, and the rapid spread of activities for the organization’s social responsibility through the media has led to a management evaluation of whether the company practices social responsibility. In addition to enterprises, healthcare organizations have also attempted to implement policy efforts to practice social responsibility as a public management strategy [8].

Nursing organizations elsewhere have also been practicing social responsibility [9,10,11], but the concept of social responsibility itself is unfamiliar to nursing organizations who lack an awareness of it. Social responsibility is also an essential concept in nursing organizations from the individual to organizational level [12,13], and it is necessary to clearly define the concept of social responsibility in nursing organizations to carry out relevant organizational activities.

Concepts are the main factors that include the attributes that describe phenomena and provide important ideas in developing nursing theory, providing a new perspective for theorists, researchers, and practitioners to explain and observe the phenomena of interest as concepts [14]. It is essential to go through the concept analysis phase to define the concept clearly [15]. Conceptual analysis clarifies the meaning of ambiguous concepts by clarifying the nature of the phenomena of interest [16]. The operational definition lists attributes and prerequisites resulting from the conceptual analysis, allows researchers to understand the basic properties of concepts, and clarifies the relationships between concepts [14]. Thus, this study applied the concept analysis method reported by Walker and Avant [14] to identify its attributes systematically through the concept analysis of social responsibility in nursing organizations and clarify the operational definition of social responsibility concepts so that others who later use the concept can use the same meaning. Therefore, this study will help promote an understanding of the concepts of social responsibility and use this to educate nursing practitioners and researchers, including nursing organizations interested in social responsibility.

## 2. Purpose of Research

The purpose of this study was to provide basic data for the development of knowledge, focusing on the social responsibility of nursing organizations by grasping its properties and clearly understanding the concept through concept analysis.

## 3. Research Method

### 3.1. Research Design

This study evaluated the concept of the social responsibility of nursing organizations by applying the concept analysis method reported by Walker and Avant [14]. This concept analysis method does not require an observation of the site. Instead, it analyzes the relevant literature to analyze how the concept of interest is defined. Therefore, in this study, the concept analysis was conducted by first identifying the scope of social responsibility through a literature review, identifying the preceding factors and properties of the concept presented in each, and checking the results.

### 3.2. Method of Data Collection and Analysis

The literature search in this study was conducted using PubMed and the Cumulative Index of Nursing and Allied Health Literature (CINAHL) for foreign literature outside of Korea. The period was set from 1990 to 2017. The search terms were ‘Social Responsibility’ AND ‘nursing’ [MeSH]. As a result, 537 articles were located in PubMed, and 386 articles were located in CINAHL. The search for domestic literature involved the academic journals and dissertations of RISS (www.riss4u.net, accessed on 21 February 2018) and DBpia (www.dbpia.co.kr, accessed on 21 February 2018); the search terms were ‘social responsibility’ and ‘social response’ and ‘nursing’. Sixty-eight articles were found in RISS and 20 in DBpia. Finally, 11 articles were assessed for the study. Based on the literature review, this study was performed according to the concept analysis procedure reported by Walker and Avant [14], and its specific eight-step analysis process is as follows:

1. Select a concept.
2. Establish the purpose of conceptual analysis.
3. Confirm all use of concepts.
4. Identify the defining attributes of a concept.
5. Present a model case of concept.
6. Provide additional examples of concepts (similar cases, opposite cases, related cases).
7. Identify the factors and consequences that predate the concept.
8. Identify empirical references to concepts

### 3.3. Ethical Consideration

This study was a review and did not require approval from the Institutional Review Board or Ethical Committee.

## 4. Research Results

### 4.1. Selection of Concepts

There is a growing need for social responsibility in healthcare organizations within the health and medical environment. Among them, the practice of social responsibility in nursing organizations has become increasingly important. Thus, the concept was selected to clarify the concept of social responsibility in nursing organizations and to identify its leading factors.

### 4.2. Purpose of Concept Analysis

The purpose of this study was to clarify the concept through the concept analysis of the social responsibility of nursing organizations, identify the leading factors and properties, and provide the basic data necessary for the practice of social responsibility of the nursing organization.

### 4.3. Confirm All Use of Concepts

#### 4.3.1. Dictionary Definition

The concept of social responsibility was not defined solely in advance. A separate search of the two words in the Webster dictionary revealed a definition of ‘social’ as ‘a duty that is usually associated with an individual or society’ and ‘responsibility’ as ‘a duty that is required and expected of an individual or organization’. According to a search in the Hankyung Economic Terms Dictionary, it is defined as “a sense of responsibility for the impact of companies on society and the environment as members of society and taking the lead in transparent management and service.” Therefore, the dictionary definition of social responsibility is interpreted as ‘a consciousness or mission related to society that requires and expects from individuals or organizations.

#### 4.3.2. Use of Concepts in Literature

The definition of social responsibility is diverse and has been identified mainly in business. In the OECD (Organization for Economic Cooperation and Development), companies take action to mature and develop symbiotic relationships between companies and society [17]. The International Chamber of Commerce (ICC) defined it as a voluntary willingness for an enterprise to engage in business responsibly [18]. The ISO defines an organization as a balanced approach to international behavior that affects society and the environment through transparent and ethical activities, seeking sustainable development that meets stakeholders’ expectations and contributes to society, health, and welfare [6]. In addition, in the business area, each scholar defined social responsibility in various terms. Barnett [19] defined good activities to develop a beautiful society, and Gariga and Mele [20] defined activities to manage various public social needs. Dahlsrud [21] stated that corporate profit-seeking is a concept that contrasts with the existing values of a company, a voluntary effort by the company to meet the needs and expectations of society, and that it is not limited to corporate social contribution or donation activities. Instead, it specifies the role and responsibility in the complex dimensions and areas that consider the comprehensive influence of the enterprise in the modern capitalist society.

#### 4.3.3. Use of Concepts in Nursing Literature (Table 1)

At the same time, this is generally evidenced by the fact that the concept of social responsibility has already been very strongly melted within the value of nursing professionals and that the basis of nursing, social responsibility, has already been practiced by historical figures representing nursing, such as ‘Florence Nightingale’ and ‘Lavinia Lilian Wald’ [11]. Defining the social responsibility of a nursing organization apart from the social justice concept is difficult. Pamela et al. [9] reported that the social responsibility of nursing is also considered in terms of social structure, such as human rights, service, poverty, and safety, as a concept that includes social justice. Reimer-Kirkham et al. [10] said that when nursing students conducted clinical practice in correctional facilities or community vulnerable groups, they had an experience of understanding the world in a new way called social justice as social issues, inequality, poverty, and their interest in the underprivileged increased. Experiencing this social justice and social participation is an important learning experience that leads to a sense of social responsibility in the field of nursing professionals [11].

**Table 1:** Definition of social responsibility of nursing organization in literature review.

Author (Year)	Definition
Kelley (2008) [1]	Social responsibility is the obligation to promote equity, access, and justice.
Belknap (2008) [2]	Social responsibility is a pedagogy of engagement that provides an effective strategy for exploring issues of race, class, gender, and structural inequalities that underlie health disparities.
Falk-Rafael (2005) [4]	Social responsibility of nursing is caring for humanity and the environment.
Mayo (1996) [5]	Social responsibility is instilled in moral and professional practice obligations and cultural sensitivity.
Pamela (2012) [9]	Social justice is asserted as a nursing responsibility.
Reimer-Kirkham (2005) [10]	Social responsibility is the commitment to issues pertaining to diversity, vulnerable populations, social determinants of health, advocacy and activism, and social justice.
Redman (2005) [11]	Social responsibility promotes nursing curriculum through service learning
Drevdahl (2001) [21]	Social responsibility is reinvesting in social justice as a capital idea for public health nursing.
Boutain (2005) [22]	Social responsibility is caring for the vulnerable.
Riley (2010) [23]	Social responsibility is described in public service as a profession.
Farcett (2005) [24]	Social responsibility is to promote social justice in global health nursing.

### 4.4. Identify the Defining Attributes of a Concept

Conceptual attributes of social responsibility, which are used in various ways in the literature, have been identified. The social responsibility of nursing organizations is the responsibility of the nursing profession on social issues, such as health-related social policies, activities, justice, human rights, inequality, poverty, and education. The conceptual attributes identified from these definitions are as follows (Table 2).

1. Access and planning for the socially oppressed and afflicted weak serve to change the nursing organization’s perception of social responsibility.
2. Educating and developing interventions to promote the practice of social responsibility in nursing organizations.
3. Praxis nursing policy approaches and strategies on social structural inequality are necessary for nursing organizations to carry out their social responsibilities continuously.

### 4.5. Model Case of Concept

The model case refers to an example that includes all of the main attributes of the concept and is based on three attributes for the social responsibility of the nursing organization: planning, action, and policy approach strategy. The model case is organized as follows.

When the Sewol ferry disaster occurred in South Korea, nursing organizations wondered how they could join in the national disaster situation. They thoroughly planned what they had to do for this. First, they organized a volunteer organization centered on the nurses’ association in the area to deliver medical assistance and necessary relief supplies to those affected by the disaster. It also showed a nursing organization working closely with the local community by continuing its strategic approach, such as reinforcing human resources and equipment at the local mental health center so that they can continue to overcome mental damage. This model makes plans for care by nursing organizations to fulfill their social responsibilities and puts them into action. Furthermore, it is a model case that includes all the attributes that try to practice social responsibility at the nursing organization level, such as ensuring that a system is established to ensure that responsible activities are sustainable.

### 4.6. Additional Cases of Concepts (Similar Cases, Opposite Cases, and Related Cases)

#### 4.6.1. Similar Cases

After the MERS outbreak in South Korea, nursing organizations reviewed and planned what they should do to protect the people’s health. They reviewed and planned what should be done by the nursing organization to protect the health of the people. They selected special personnel for rapid suppression of the MERS outbreak and placed them in major hospitals. As a result, the dedication and hard work of frontline nurses and many medical staff quickly brought MERS under control. It also embodied systematic strategies, including establishing a system to prepare for infectious diseases, such as MERS, at all times in the future. 

Subsequently, the government encouraged the hard-working medical staff with proper compensation, but the hard-working nurses became frustrated when they belatedly learned that the most hard-working nurses were not given proper compensation and treatment. In this similar case, the nursing organization devoted itself to fulfilling its social responsibilities in the health and medical environment. It made efforts to establish a strategic response system for post-care. Nevertheless, the situation in which hard-working nurses in the nursing organization were excluded from proper treatment and compensation is a case in which the nursing organization failed to take care of them until the end, and the nature of the care plan, like social responsibility, was not included.

#### 4.6.2. Opposite Cases

One day, nursing organization received an urgent request for help from a nurse who works as a local female lawmaker. She said she had endured sexual harassment and sexual insults often in a council composed of mostly males. She could not endure this treatment anymore, so she turned to nursing organizations for help. The nursing organization began to talk to her but refused to give a statement of support because she was not currently a nurse working in a hospital. In response, she reiterated her request, saying that she had never forgotten her identity as a nurse and that she was now a member of the National Assembly. The nursing organization reluctantly said yes, but she did not have a thorough plan or strategy to resolve the situation. Even when the Council Ethics Committee was convened to determine the truth, the nursing organization did not participate and only ordered her subordinates to show their faces. This is a case in which nursing organizations do not include any attributes of social issues, such as sexual harassment, care plans, actions, and policy strategies for sexual harassment.

#### 4.6.3. Related Cases

The nursing organization has protested against the government in legal struggle issues. They attended the candlelight rally in cities every week with great enthusiasm. Although they had been scheduled to work the next day, they attended the rally until the dawn of the previous day. As a result, there were cases where medical accidents almost occurred because of their neglect of patient care while on duty. In this case, the excessive participation in rallies has instead raised concerns about medical accidents in hospitals and did not include care planning and policy approaches, an unplanned and eager attribute of social responsibility. Furthermore, it is difficult to say that taking care of patients in hospitals is also a proper practice of social responsibility because it has been overlooked that it is a responsibility to be taken care of as a nursing organization.

### 4.7. Identify the Antecedents and Consequences of a Concept

In this process, the antecedents are an event or incidental condition that happens before a concept occurs, and the consequences are an event or condition that takes place after the concept [14].

#### 4.7.1. Antecedents

The first leading antecedent in the concept of social responsibility in nursing organizations identified in the literature is the perception of social issues. Recognizing is acknowledging the need for action [20], which can be described as a key antecedent that clearly indicates the need for social responsibility in nursing organizations. The second antecedent factor is participation experience. This is because they practice positive social responsibility behaviors by participating in community service at the healthcare organization level and performing nursing for the socially vulnerable [11].

#### 4.7.2. Checking the Consequences

**Figure 1:**
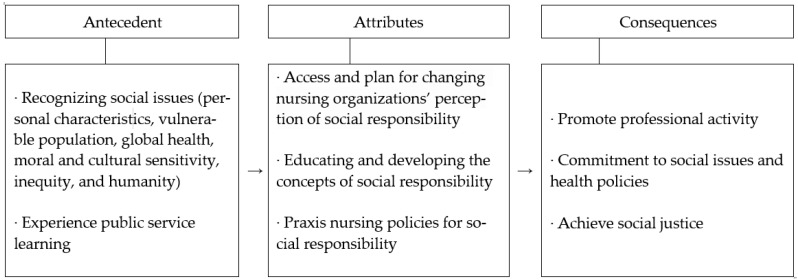
Conceptual structure of social responsibility of nursing organizations.

Recognizing the need for caring for social issues, this study confirmed from the start of the participation experience that it was the social responsibility of the nursing organization to promote, commit, and achieve a policy approach for continuous participation in social issues, such as health-related policies, social justice, human rights and inequality as a group of health experts. Thus, the conceptual framework embodied based on the social responsibility of the nursing organization is the same as in Figure 1.

### 4.8. Confirm Conceptual Empirical Reference

The empirical reference is the final stage of concept analysis that confirms that the attribute of concept exists in the phenomenon and clarifies the vague concept used in clinical practice, which has not been conceptualized. In addition, if the attribute the tool is trying to measure in the research design reflects the attribute shown in the conceptual analysis well, it can be used as an empirical reference. As an empirical reference to the social responsibility of the nursing organization identified through these studies [1,2,3,4,5,9,10,11], these articles analyzed the social responsibility in nursing organizations, such as nursing schools, hospitals, and nursing institutions, is a very strong core value for the development and promotion of the nursing profession. This is because nursing organizations must fulfill their social responsibilities for human rights and ethical dilemmas they face and strategically approach in the rapidly changing medical environment.

## 5. Discussion

This study analyzed the concept of social responsibility in nursing organizations using the concept analysis method reported by Walker and Avant [14]. This method has not been used to provide a clear meaning of social responsibility, which is used widely in other areas. Therefore, this study attempted to clarify its conceptual properties and use them actively later as concepts of clear meaning. In this study, the first attribute of the concept of social responsibility of nursing organizations was derived: ‘Access and Plan for caring’, which must be preceded to practice behavior effectively [22,23]. Access and Planning includes a specific strategy for carrying out the social responsibilities of the nursing organization, as well as an approach for situations that cannot be carried out [24]. This care plan should be preceded by a clear awareness of human views, professional values, competencies, activities, and problem-solving-oriented nursing [12]. Therefore, it would be necessary to consider the need for agreed strategies of leaders in nursing organizations to establish care plans for social responsibility through this recognition and to ensure that planned actions are carried out.

The second attribute is educating and developing concepts of social responsibility through nursing curricula and service learning. Putting a plan into action can be challenging. Thus, educating and developing the social responsibilities of nursing organizations while continuing to provide opportunities for participation experience will play an essential role in developing care plans into action [25,26]. In particular, educational strategic access, which means socially responsive education, includes what education contains concerning the value of social responsibility and how education is conducted [1,27,28]. Therefore, education on social responsibility at the site where various nursing education is conducted is a very important attribute related directly to behavior. Hence, a systematic approach at the nursing organization level will be needed to ensure that this education is conducted properly. The third attribute is praxis nursing policy strategies on social structural inequality in nursing organizations to carry out their social responsibilities continuously. Based on the results of this study, the social responsibility concept of nursing organization can be used clearly through social responsibility recognition and behavior and nursing policy approaches that are important in the preceding factors and attributes of social responsibility of nursing while also enhancing the social responsibility of nursing organization through program development and application for this.

Finally, this study has some limitations. Although it attempted to analyze the published indexed articles comprehensively, some publications may have been missed. In addition, the lack of search for literature on social justice of organizational dimensions, which is also being used in the concept of social responsibility, could also be a limitation of this study. Therefore, future studies should examine the various attributes and related factors of the concept of social responsibility of nursing organizations that have not been identified in this study.

## 6. Conclusions

This study analyzed the concept of social responsibility in nursing organizations using the concept analysis method reported by Walker and Avant [14]. The concept of social responsibility of the nursing organization derived from this study was identified as a care plan for issues related to social issues surrounding nursing and a property that leads to a policy approach through practice. These results will help develop an environment in which social responsibility values are delivered at all sites of the nursing organization and that more social responsibilities given to the nursing organization can be fulfilled by continuously educating, performing, and evaluating the recognition and practice of social responsibility at the level of nursing college students, nurses, and nursing organizations.

## Figures and Tables

**Table 2:** Antecedent, attributes, and consequences of social responsibility of nursing organization in literature review.

Author (Year)	Antecedent	Attributes	Consequences
Kelley (2008) [1]	Understanding obligation	Access equity and justice	Promote social responsibility
Belknap (2008) [2]	Recognizing social issues	Education for engagement	Care for health disparities
Falk-Rafael (2005) [4]	Understanding of inequity	Legacy of political action	Critical caring for human rights and the environment
Mayo (1996) [5]	Recognizing moral and cultural sensitivity	Explore effective strategies for social issues	Promote social responsibility
Pamela (2012) [9]	Understanding injustice	Structuring nursing knowledge development and policy initiatives	Support of essential goals
Reimer-Kirkham (2005) [10]	Understanding of inequity environment	Learning social justice and activism in nursing curricula	Commitment to social and political mandates
Redman (2005) [11]	Experience service learning	Learning about the responsibility of citizenship	Promote nursing curriculum through service learning
Drevdahl (2001) [21]	Understanding ethical principle	developing community interventions	Reengagement in Public health activities
Boutain (2005) [22]	Understanding of societal inequality	Teaching concepts of Health disparity	Care for the vulnerable
Riley (2010) [23]	Experience Public service	Public service Action	Promote profession activity
Farcett (2005) [24]	Analysis of organizational policies	Access to health care policies	Justice and social change

## Data Availability

Data is contained within the article.

## Public Involvement Statement

No public involvement in any aspect of this research.

## Guidelines and Standards Statement

This manuscript was drafted against the PRISMA [29] for systematic reviews research.
